# Development of a Prognostic Alternative Splicing Signature Associated With Tumor Microenvironment Immune Profiles in Lung Adenocarcinoma

**DOI:** 10.3389/fonc.2022.880478

**Published:** 2022-06-27

**Authors:** Guangyao Bao, Tian Li, Xiaojiao Guan, Yao Yao, Jie Liang, Yifan Xiang, Xinwen Zhong

**Affiliations:** ^1^ Department of Thoracic Surgery, First Affiliated Hospital, China Medical University, Shenyang, China; ^2^ School of Basic Medicine, Fourth Military Medical University, Xi’an, China; ^3^ Department of Pathology, Shengjing Hospital, China Medical University, Shenyang, China

**Keywords:** alternative splicing, lung adenocarcinoma, prognosis, immune response, immune microenvironment

## Abstract

**Background:**

Alternative splicing (AS), a pivotal post-transcriptional process across more than 95% of human transcripts, is involved in transcript structural variations and protein complexity. Clinical implications of AS events and their interaction with tumor immunity were systematically analyzed in lung adenocarcinoma (LUAD).

**Methods:**

Transcriptome profiling as well as AS data of LUAD were retrospectively curated. Then, the network of the overall survival (OS)-relevant AS events with splicing factors was established. After screening OS-relevant AS events, a LASSO prognostic model was conducted and evaluated with ROC curves. A nomogram that integrated independent prognostic indicators was created. Immune response and immune cell infiltration were estimated with ESTIMATE, CIBERSORT, and ssGSEA algorithms. Drug sensitivity was inferred with pRRophetic package.

**Results:**

In total, 2415 OS-relevant AS events were identified across LUAD patients. The interaction network of splicing factors with OS-relevant AS events uncovered the underlying regulatory mechanisms of AS events in LUAD. Thereafter, a prognostic model containing 12 AS events was developed, which acted as a reliable and independent prognostic indicator following verification. A nomogram that constituted stage and risk score displayed great effectiveness in evaluating the survival likelihood. Moreover, the AS-based prognostic model was in relation to immune response and immune cell infiltration. Patients with a high-risk score displayed therapeutic superiority to cisplatin, erlotinib, gefitinib, and gemcitabine. Finally, three AS-relevant genes (CDKN2A, TTC39C, and PKIB) were identified as prognostic markers.

**Conclusion:**

Collectively, our findings developed an AS event signature with powerful prognostic predictive efficacy in LUAD.

## Introduction

As the highest incidence cancer type, lung cancer also causes the most cancer-related deaths (1, 2). According to reports, 85% of all new lung cancers each year are non-small cell lung cancer (NSCLC), with a dismal 5-year survival rate of < 16% (3). Currently, lung adenocarcinoma (LUAD) accounts for the leading pathological subtype of NSCLC, which exhibits rising morbidity among young women and non-smokers (4). Moreover, patients with advanced lung adenocarcinoma are often accompanied by poor long-term prognosis. Currently, surgical resection plus radio- or chemotherapy represents the first choice and main therapeutic means against LUAD (5, 6). Despite recent advances in immunotherapeutic strategies, LUAD patients display diverse responses to immune-based therapies (6–8). Few schemes to prevent and early treat LUAD are developed mainly because of the few characteristic targets upon molecular pathogenesis (9).

Alternative splicing (AS), a pervasive cellular process, exerts a critical function in the post-transcriptional process where a variety of transcripts from the same gene are generated, contributing to proteome complexity (10). More than 95% of human genes incur AS events during physiological process (11). AS events are remarkedly modulated with tissue and developmental stage-specific manners, which are often deregulated in diverse cancer types (12). Abnormal RNA splicing drives tumor initiation and progression through affecting metabolic reprogramming, proliferation, metastases, and resistance of tumor cells and microenvironment (13–16). Moreover, deregulated splice variants produce effects on the therapeutic responses to targeted therapy, radio-, chemo- and immunotherapies (17). Thus, it is mostly important to ascertain pathological splicing isoforms regarding the development of novel practical markers and clarifying the mechanisms involving in deregulated AS events, eventually expounding the influences on cancers, and offering more effective treatment schemes. To date, accumulated evidence uncovers the biological relevance as well as clinical implications of AS events during lung tumorigenesis (18–21). Lung carcinogenesis principally evolves by sequential genetic changes and genomic deregulation, which is also influenced by tumor microenvironment. LUAD exhibits interpatient and intratumor heterogeneity in tumor cells and microenvironment (22). Nevertheless, the underlying relations of AS events with tumor microenvironment of LUAD remain ill-defined.

Herein, our research conducted comprehensive analyses upon AS events across LUAD and identified LUAD-specific AS events for developing novel prognostic markers. Moreover, our findings provided novel thinking about the interactions between AS events and immunity in LUAD.

## Materials and Methods

### Data Retrieval

Transcriptome profiling and clinicopathologic characteristics of 522 LUAD specimens were retrospectively curated from the Cancer Genome Atlas (TCGA) project utilizing TCGAbiolinks R package (23). **Table 1** lists clinicopathological data of 522 LUAD patients. AS data were curated from TCGA SpliceSeq (https://bioinformatics.mdanderson.org/TCGASpliceSeq) (24). Then, Percent Spliced In (PSI) values that ranged from 0 to 1 were determined for AS events across transcripts. AS events were classified into seven forms, containing Alternate Donor site (AD), Alternate Acceptor site (AA), Alternate Terminator (AT), Alternate Promoter (AP), Mutually Exclusive Exons (ME), Exon Skip (ES), and Retained Intron (RI). AS events with PSI value ≥ 75%, and average PSI value ≥ 0.05 were enrolled for subsequent analysis. UpSetR package was employed for visualizing the distribution of AS events in LUAD (25).

**Table 1:** Clinicopathological characteristics of 522 LUAD patients from TCGA cohort.

Characteristics	Type	n	Proportion (%)
Age	≤65 > 65 unknown	241 262 19	46.2 50.2 3.6
Gender	Female Male	280 242	53.6 46.4
Stage	I-II III-IV unknown	403 111 8	77.2 21.3 1.5
T stage	T1-2 T3-4 unknown	453 66 3	86.8 12.6 0.6
N stage	N0-1 N2-3 unknown	433 77 12	83.0 14.8 2.2
M stage	M0 M1 unknown	353 25 144	67.6 4.8 27.6

### Screening OS-Relevant AS Events in LUAD

OS-relevant AS events were selected across LUAD patients through the survival R package utilizing univariate regression analyses following the criteria of p-value < 0.05. In addition, UpSet and volcano plot were adopted for describing the distribution of OS-relevant AS events. Thereafter, the first 20 AS events in different types of AS were visualized into bubble plots.

### Establishment of an OS-Relevant Splicing Factor-AS Interaction Network

SpliceAid project was employed to curate specific splicing factors (26). Furthermore, Pearson correlation test was adopted for analyzing the interactions of splicing factors with OS-relevant AS events. The Cytoscape (version 3.8.0) was utilized for visualizing this interactional network of splicing factors with OS-relevant AS events and correlation coefficient > 0.6 as well as p < 0.05 as the filtering criteria (27).

### Construction and Validation of Predictive Models Based on AS Events

The glmnet R package was adopted to establish a least absolute shrinkage and selection operator (LASSO) prognostic model based on OS-relevant AS events across LUAD patients (28). The prognostic scoring formula was conducted with this formula: risk score= PSI value of AS event1 × Coef1 + PSI value of AS event2 × Coef2 … + PSI value of AS eventn × Coefn, in which Coefn represented the regression coefficient. Then, we stratified LUAD patients into different risk subpopulations according to median risk score. The receiver operating characteristic (ROC) curve was generated utilizing timeROC R package for showing the specificity and sensitivity of risk score in evaluating prognosis of LUAD. The Kaplan-Meier curves were applied to assess the differences in OS rate with the survival R package. Additionally, Cox regression models were conducted for analyzing the interactions of age, gender, tumor stage, and risk score with OS outcomes.

### Construction of a Prognostic Nomogram

In order to evaluate OS outcomes, a prognostic nomogram comprised of independently prognostic indicators AS-relevant risk signature as well as stage was conducted for estimating 1‐, 2‐, and 3‐year OS probabilities with the rms R package. Subsequently, calibration curves which showed the survival implications of this nomogram were depicted. The calibration curve close to 45° was considered as an excellent indicator in this nomogram.

### Immune Cell Infiltrations Estimated *via* Deconvolution Algorithm and Single-Sample Gene Set Enrichment Analysis (ssGSEA)

The cell type identification by Estimating Relative Subsets Of RNA Transcripts (CIBERSORT) deconvolution algorithm was adopted to estimate the abundances of 22 diverse leukocyte subsets (29). CIBERSORT results for samples with p < 0.05 indicated that the estimated abundances of leukocyte subsets were reliable, which were eligible for subsequent analysis. For each specimen, estimations were standardized to sum up to 1, thereby being interpreted directly as cellular fraction. The ssGSEA from Gene Set Variation Analysis (GSVA) was employed for quantification of the relative abundances of 29 immune cells as well as functions following the special feature gene panels across LUAD specimens (30). The ssGSEA enrichment score was indicative of the relative abundance, which was standardized to range from 0 to 1.

### Identifying and Comparing the Immune Profiles of Different Risk Groups

The Estimation of Stromal and Immune Cells in Malignant Tumors using Expression Data (ESTIMATE) R package possesses the significant advantage in estimating the specific features of transcriptome profiles (31). The gene sets of immune checkpoints were downloaded from recent research (32, 33). The mRNA expression of immune checkpoints was quantified across LUAD specimens. Tumor mutational burden (TMB) was employed to predict clinical response to immunotherapy (34). TMB was calculated according to the formula: (entire counts of variants)/(the entire lengths of exons) in line with the variants of LUAD specimens that were extracted from the mutational profiles.

### Estimation of Drug Sensitivity

Half-maximal inhibitory concentration (IC50) values for cisplatin, gemcitabine, gefitinib, and erlotinib were estimated with the pRRophetic R package by ridge regression analysis (35, 36). IC50 indicated the treatment response to above chemotherapeutic agents in TCGA cohort.

### Statistical Analysis

Spearman’s correlation analysis was conducted to estimate composition differences. Wilcoxon signed rank test was applied for comparisons in two groups. Kaplan-Meier survival curve was implemented for evaluating the survival differences between groups. Cox regression analysis was conducted for verifying the associations of certain indicators with LUAD prognosis. To evaluate the performance of prognosis prediction, time-independent ROC curves were conducted and area under the curve (AUC) was calculated with timeROC R package. Statistical analysis was achieved utilizing R software (version 4.02). P <0.05 was taken into consideration statistically.

## Results

### Identification of OS-Relevant AS Events in LUAD

**Figure 1:**
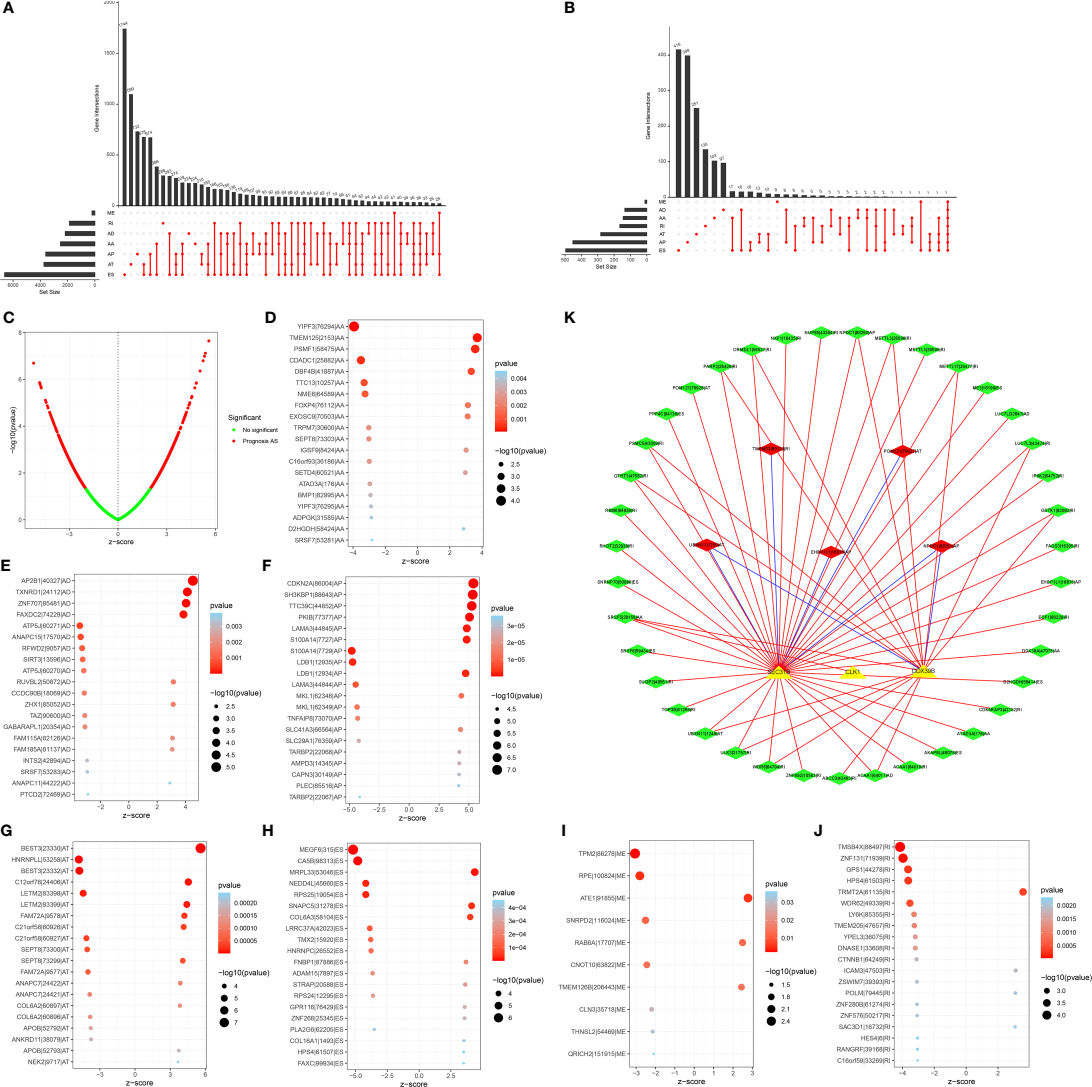
Prognosis-relevant AS events and their interactions with splicing factors across LUAD patients. **(A)** UpSet plot showing numbers and percentages of AS events as well as their interactions across LUAD specimens. **(B)** Volcano plots of OS-relevant AS events in LUAD. Red dots represented AS events that were distinctly correlated to OS, but green dots did not affect patients’ OS. **(C)** UpSet showing numbers and percentages of seven types of OS-relevant AS events and their interactions in LUAD. **(D–J)** Bubble plots of the distribution of the first 20 most significant AS events in LUAD, which indicated that the seven alternative splicing modes exhibit great variability. **(K)** The OS-relevant splicing factor-AS interaction network in LUAD. Triangle bubbles indicated splicing factors and diamond bubbles indicated AS events. The red and blue line separately indicated positive and negative connection in splicing factors and AS events. Red and green diamond bubbles separately meant adverse and favorable prognosis-relevant AS events.

In total, 43,945 AS events were identified across 522 LUAD patients (**Figure 1A**). ES accounted for the most frequent AS signature, followed by AT and AP. Univariate analyses were presented to qualify the impact of each AS event on patients’ OS. Subsequently, 2415 AS events displayed remarked associations with survival outcomes of LUAD patients, in which 1356 were protective factors and 1059 were risk factors (**Figure 1B**). Notably, one gene may possess two or more OS-relevant AS events across LUAD patients, as shown in the UpSet plots (**Figure 1C**). The first 20 significant OS-relevant genes of AS events are separately shown in **Figures 1D–J**, which indicated that the seven alternative splicing modes exhibit great variability.

### Construction of an OS-Relevant Splicing Factor-AS Interaction Network in LUAD

Splicing factors act as dominant regulators of AS events, which may affect the splicing of oncogenes as well as tumor suppressors (37). For exploring the underlying interactions of the expressions of splicing factors with AS events, we visualized the splicing-regulatory network, as depicted in **Figure 1K**. In total, three splicing factors (including SEC31B, CLK1, and DDX39B) displayed prominent associations with 44 OS-relevant AS events. Furthermore, most favorable AS events exhibited positive interactions with the expression of splicing factors and the three splicing factors were in relation to multiple AS events. Thus, splicing factors may act as an indispensable role in modulating AS events during lung carcinogenesis.

### Development of a Reliable Prognostic AS Event-Based Signature in LUAD

**Figure 2:**
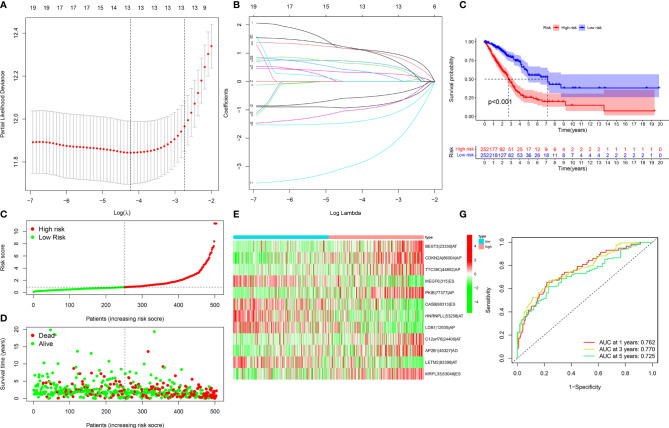
Development of a reliable prognostic model for LUAD patients. **(A)** The distribution of partial likelihood deviance corresponding to λ-logarithm value. **(B)** LASSO coefficient profiling of OS-relevant AS events. The lines stood for OS-relevant AS events and candidates AS events were chosen utilizing ten-fold cross-verification with minimum criteria. **(C)** The distribution of risk score across LUAD patients. Red dots meant high-risk patients while green dots meant low-risk patients. **(D)** Scatter plots depicted distribution of LUAD patients’ survival time and status. Red dots denoted patients who were dead, whereas green dots denoted patients who were alive. **(E)** Heatmap displayed the distribution of PSI values for the established prognostic model. **(F)** Kaplan–Meier survival curves of high- and low-risk LUAD patients. **(G)** ROC curves at 1, 3, and 5 years of the prognostic model for LUAD patients.

For avoiding over-fitting, LASSO Cox analysis was adopted for developing a prognostic model of LUAD on the basis of OS-relevant AS events. Through cross-verification, the optimal parameters were selected (**Figure 2A**) and the coefficients in LASSO regression model were determined (**Figure 2B**). Ultimately, 12 OS-relevant AS events (BEST3|23330|AT, CDKN2A|86004|AP, TTC39C|44852|AP, MEGF6|315|ES, PKIB|77377|AP, CA5B|98313|ES, HNRNPLL|53258|AT, LDB1|12935|AP, C12orf76|24406|AT, AP2B1|40327|AD, LETM2|83398|AT, MRPL33|53046|ES) were identified (**Table 2**). In line with the regression coefficients and PSI value of 12 OS-relevant AS events, we calculated risk scores of LUAD patients. Thereafter, LUAD patients were classified into different groups with median risk score of 0.8834 (**Figure 2C**). Moreover, we noticed that high-risk subpopulations were often accompanied by high mortality (**Figure 2D**). Heatmap depicted the heterogeneity in PSI values of 12 OS-relevant AS events (**Figure 2E**). Prognostic analyses uncovered that high-risk subpopulations exhibited remarkedly dismal OS outcomes (**Figure 2F**). The validity of the prognostic model in prognosis prediction was verified through ROC analysis. The AUC values at 1-, 3-, and 5-year OS were separately 0.762, 0.770, and 0.725, showing the good effectiveness of this model in prognosis prediction (**Figure 2G**).

**Table 2:** Twelve OS-relevant AS events in the LASSO prognostic model.

AS events	Coefficient	HR	HR.95L	HR.95H	P-value
BEST3|23330|AT	1.23	3.43	1.07	10.93	0.038
CDKN2A|86004|AP	1.27	3.55	1.68	7.52	< 0.001
TTC39C|44852|AP	0.78	2.19	0.86	5.59	0.101
MEGF6|315|ES	-1.57	0.21	0.08	0.52	< 0.001
PKIB|77377|AP	0.35	1.42	0.75	2.71	0.281
CA5B|98313|ES	-0.99	0.37	0.14	0.98	0.045
HNRNPLL|53258|AT	-3.38	0.03	0.004	0.30	0.002
LDB1|12935|AP	-0.65	0.52	0.16	1.67	0.275
C12orf76|24406|AT	0.70	2.01	0.39	10.38	0.402
AP2B1|40327|AD	0.51	1.68	0.38	7.51	0.497
LETM2|83398|AT	-1.13	0.32	0.11	0.93	0.036
MRPL33|53046|ES	1.40	4.06	0.57	29.21	0.163

### Associations of the Prognostic Model With Clinicopathological Characteristics of LUAD

**Figure 3:**
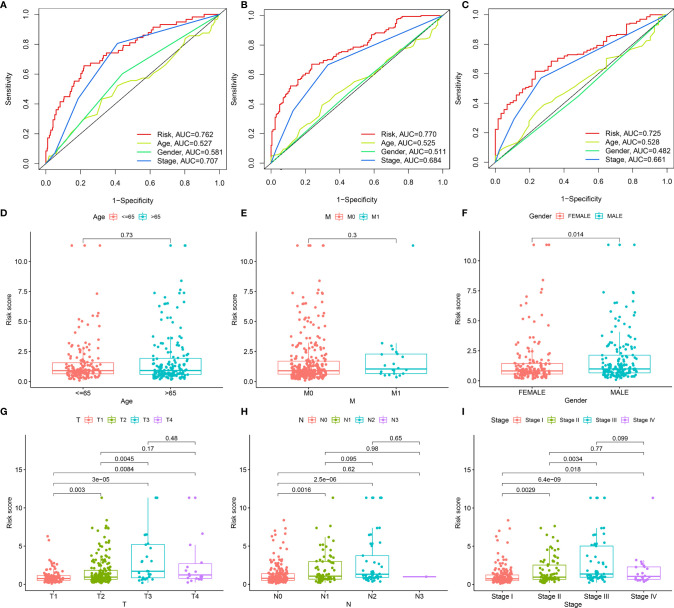
Associations of the prognostic model with clinicopathological characteristics of LUAD. **(A–C)** Comparisons of AUC at 1-, 3-, and 5-year survival estimated by risk score and clinicopathological characteristics through ROC analysis. **(D–I)** Box plots showing the distribution of risk scores in distinct clinicopathological characteristics, containing **(D)** age (≤65 vs. > 65), **(E)** M stage (M0 vs. M1), **(F)** gender (female vs. male), **(G)** T stage (T1 vs. T2 vs. T3 vs. T4), **(H)** N stage (N0 vs. N1 vs. N2 vs. N3) and **(I)** stage (stage I vs. stage II vs. stage III vs. stage IV).

Through ROC analysis, we presented the comparisons of AUC values and noticed that risk score displayed the higher AUC values under 1-, 3-, and 5-year survival compared with clinicopathological features (age, gender, and stage; **Figures 3A–C**). Additionally, the differences in risk score between distinct clinicopathological features were compared among LUAD patients. No significant differences were observed between age ≤65 and > 65 (**Figure 3D**) as well as between non-metastasis (M0) and metastasis (M1; **Figure 3E**). Increased risk score was investigated in male and female patients (**Figure 3F**). As T, N, and stage increased, risk score was gradually elevated (**Figures 3G–I**), indicating that the prognostic model contributed to LUAD progression.

### The Prognostic Model Acts as an Independently Prognostic Indicator of LUAD

**Figure 4:**
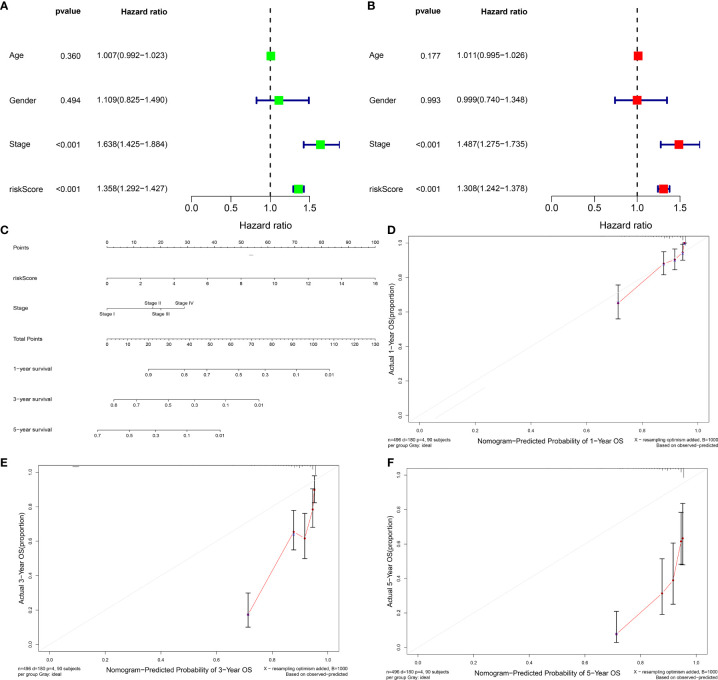
Evaluation of the independence of prognostic model in prognostic prediction and construction of prognostic nomograms for LUAD. **(A, B)** Univariate and multivariate Cox analysis of risk score and clinicopathological features with LUAD prognosis. **(C)** The nomogram of risk score signature and stage for prediction of 1-, 3-, and 5-year OS of LUAD. **(D–F)** Calibration curves used to compare nomogram estimated 1-, 3-, and 5-year survival probabilities with actual survival time.

We further verified the prognostic value of clinical characteristics and risk score and found that risk score and stage possessed the potential to independently predict LUAD prognosis (**Figures 4A, B**). Thereafter, a prognostic nomogram containing independent prognostic indicators risk score as well as clinicopathological stage was conducted for forecasting patients’ outcomes (**Figure 4C**). Calibration curves were indicative of the powerful prognostic predictive capacity of this nomogram in 1-, 3-, and 5-year OS (**Figures 4D–F**).

### Development of a Prognostic Nomogram Containing the Prognostic Model and Stage

For further applying our findings to clinical practice, this study constructed a nomogram prognostic score system in the prediction of 1-, 3-, and 5-year OS outcomes of LUAD patients (**Figure 4C**). The scoring system included the prognostic model and stage. Thereafter, for verifying the reliability of the prognostic nomogram, calibration plots were conducted and confirmed the practical significance of the model. As depicted in **Figures 4D–F**, the model possessed the potential in determining survival outcomes with a high predicted accuracy.

### Associations of the Prognostic Model With Tumor Immunity

**Figure 5:**
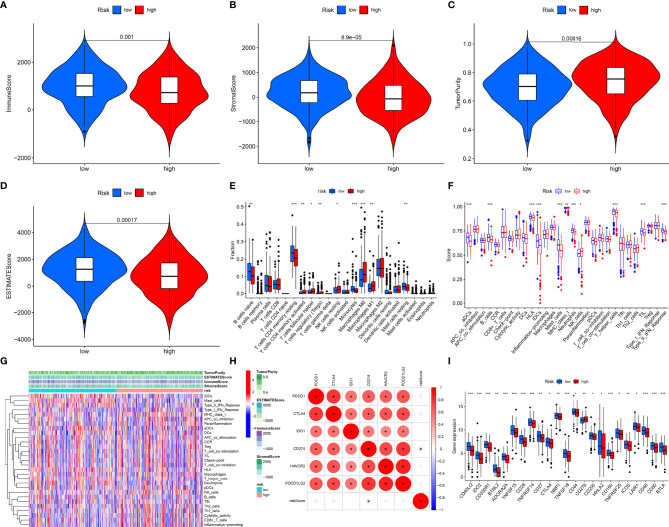
Associations of prognostic model with immune microenvironment across LUAD patients. **(A–D)** Distribution of estimate, immune, and stromal score and tumor purity in different risk groups. **(E)** Comparisons of the levels of tumor immune infiltration in different risk subpopulations with CIBERSORT algorithm. **(F)** Comparisons of the abundance levels of immune cell infiltrations and immune functions in different risk subpopulations utilizing ssGSEA algorithm. **(G)** Heatmap visualizing the distribution of the abundance levels of immune cell infiltrations and immune functions. **(H)** Associations of risk score signature and common immune checkpoint molecules across LUAD. **(I)** Comparisons of the expressions of immune checkpoint molecules in different risk subpopulations. *P < 0.05; **p < 0.01; ***p < 0.001.

We firstly estimated infiltration levels of immune and stromal cells across LUAD patients. Accordingly, patients with a high-risk score displayed reduced immune score and stromal score (**Figures 5A, B**). Nevertheless, higher tumor purity was investigated in high-risk patients (**Figure 5C**). Then, we determined ESTIMATE and noticed the prominently decreased ESTIMATE score in the high-risk group (**Figure 5D**). Thus, low-risk tumors were accompanied by abundant infiltrations of immune and stromal cells. Then, we systematically investigated the immune cell infiltration landscape across LUAD with CIBERSORT algorithm. We noticed that the low-risk group displayed high infiltration levels of B cells naïve, T cells CD4 memory resting, monocytes, and mast cells resting (**Figure 5E**). Oppositely, the high-risk group exhibited increased infiltration levels in T cells CD4 memory activated, T cells follicular helper, T cells regulatory (Tregs), NK cells resting, macrophages M0, and macrophages M1. **Supplementary Figure 1** displays interactions of risk score signature with above immune cell infiltrations. Subsequently, we revealed the activities of immune functions and immune cell infiltrations across LUAD ssGSEA method. In **Figures 5F, G**, higher abundance levels of aDCs, B cells, HLA, iDCs, mast cells, neutrophils, T helper cells, TIL, and type II IFN response were investigated in the low-risk group while MHC class I and NK cells exhibited higher abundance levels in the high-risk group. We also evaluated the interactions of the prognostic model with immune checkpoints across LUAD. As depicted in **Figure 5H**, this prognostic model possessed a positive association with CD274. Moreover, we observed that the low-risk group was characterized by increased expression of most immune checkpoint-related genes (**Figure 5I**). Thus, low-risk patients were indicative of higher immune response as well as immune cell infiltration.

### Associations of the Prognostic Model With TMB and Drug Responses

**Figure 6:**
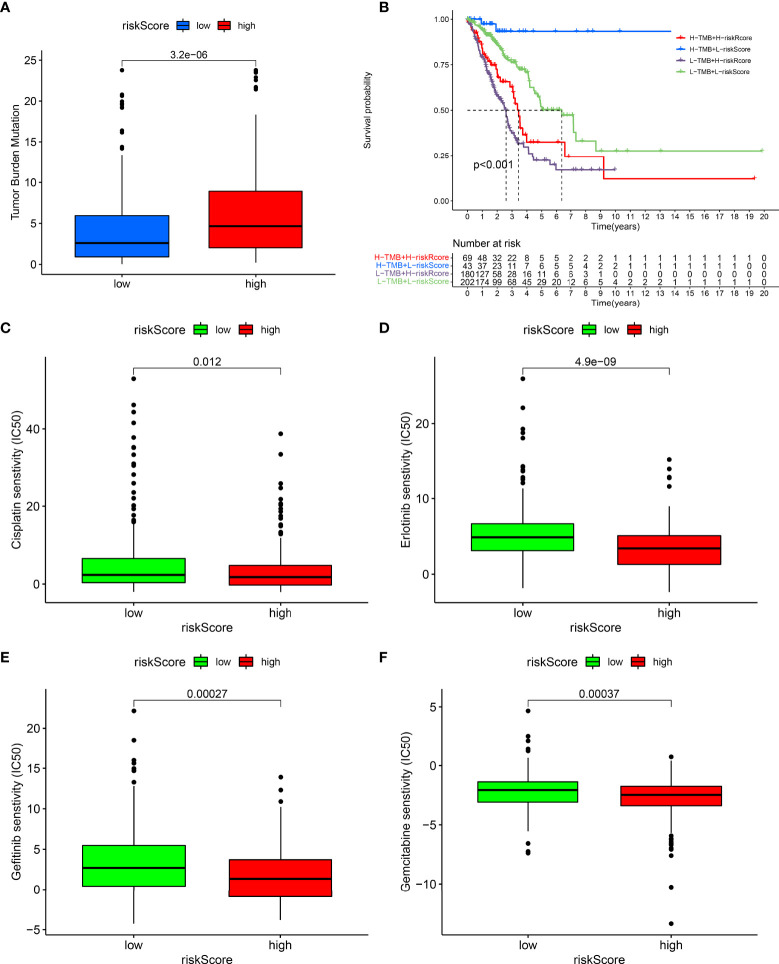
Associations of prognostic model with TMB and drug responses. **(A)** Comparisons of TMB score between high- and low-risk groups. **(B)** Survival analysis of different TMB score and risk score groups. **(C–F)** Comparisons of sensitivity to cisplatin, erlotinib, gefitinib, and gemcitabine between high- and low-risk groups.

The interaction of the prognostic model with TMB was also observed across LUAD. As shown in **Figure 6A**, the high-risk group exhibited a remarkedly increased TMB score. Moreover, we presented survival analysis among diverse subgroups. We noticed that subpopulations possessing an elevated TMB score as well as a reduced risk score displayed the most favorable survival outcomes while those with a low TMB score and high-risk score exhibited the poorest survival outcomes (**Figure 6B**). Chemotherapy and targeted therapy were gradually applied in treatments for patients with advanced lung adenocarcinoma. It is of great significance to evaluate the responses of certain drugs in different risk subpopulations. Herein, we identified the treatment responses of some drugs that were widely used in the treatment of LUAD. As shown in **Figures 6C–F**, the high-risk group possessed prominently lowered IC50 values of cisplatin, erlotinib, gefitinib, and gemcitabine, indicating that this subpopulation possessed higher sensitivity to these therapeutic agents. The above findings provide more clues for individualized treatment strategies in LUAD patients.

### Identification of Prognostic AS Events-Related Genes

**Figure 7:**
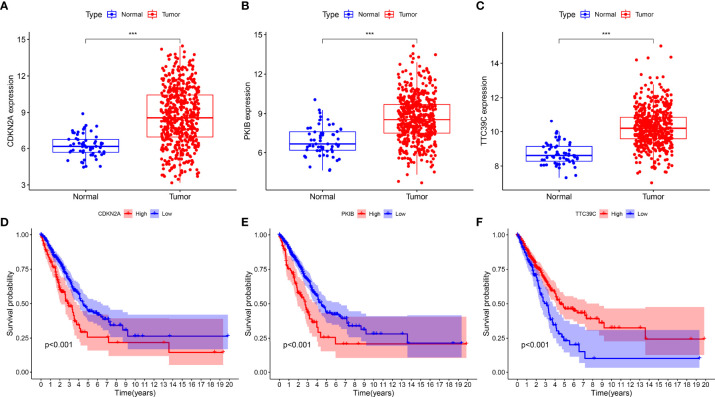
Identification of prognostic AS events-related genes. **(A–C)** The expression patterns of CDKN2A, PKIB, and TTC39C in LUAD and normal tissues. **(D–F)** Kaplan-Meier plots of different expression of CDKN2A, PKIB, and TTC39C. ***p < 0.001.

We found that CDKN2A, PKIB, and TTC39C exhibited a higher expression in LUAD than normal tissues among the 12 AS events-relevant genes in the prognostic models (**Figures 7A–C**). Moreover, survival analysis uncovered that highly expressed CDKN2A and PKIB were in relation to more dismal survival probabilities of LUAD (**Figures 7D, E**). In contrast, high TTC39C expression was indicative of the marked survival advantage (**Figure 7F**).

### Associations of Prognostic AS Events-Related Genes With Immune Microenvironment

**Figure 8:**
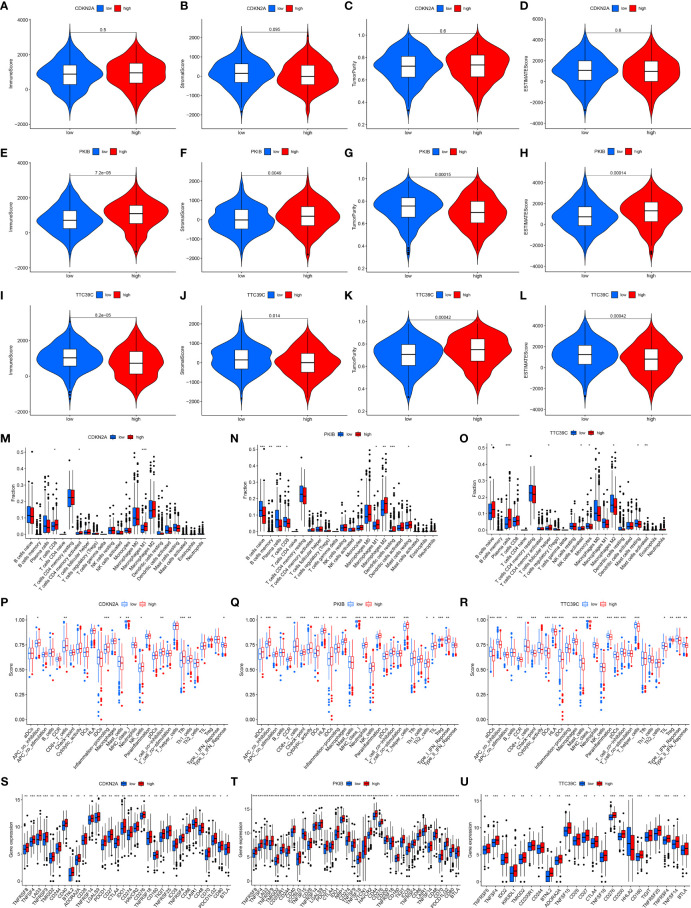
Associations of prognostic AS events-related genes with immune response and immune cell infiltration. **(A–D)** Violin plots depicted the distribution of estimate, immune, and stromal score as well as tumor purity in high and low CDKN2A groups. **(E–H)** Violin plots depicted the distribution of estimate, immune, and stromal score as well as tumor purity in high and low PKIB groups. **(I–L)** Violin plots depicted the distribution of estimate, immune, and stromal score as well as tumor purity in high and low TTC39C groups. **(M–O)** The distribution of the abundance levels of tumor-infiltrating immune subpopulations in high and low expression of CDKN2A, PKIB, and TTC39C groups. **(P–R)** The distribution of the abundance levels of immune cell infiltrations and immune functions in high and low expression of CDKN2A, PKIB, and TTC39C groups. **(S–U)** Expression levels of immune checkpoint related genes in high and low CDKN2A, PKIB, and TTC39C subpopulations. *P < 0.05; **p < 0.01; ***p < 0.001.

We further investigated the interactions of the three prognostics AS events-related genes (CDKN2A, PKIB, and TTC39C) with immune response and immune cell infiltration across LUAD. We found that deregulated CDKN2A did not affect estimate, immune, and stromal score as well as tumor purity (**Figures 8A–D**). For **Figures 8E–H**, high PKIB expression was characterized by increased estimate, immune, and stromal score but reduced tumor purity. Moreover, high TTC39C expression displayed remarkedly decreased estimate, immune, and stromal score but elevated tumor purity (**Figures 8I–L**). In **Figure 8M**, CDKN2A upregulation was in relation to increased infiltration levels of T cells CD8, T cells CD4 memory activated, and macrophages M1. PKIB deregulation was in relation to infiltrations of B cell naïve, B cells memory, plasma cells, T cells CD8, macrophages M1, macrophages M2, dendritic cells resting and mast cells resting (**Figure 8N**). B cells native, plasma cells, T cells follicular helper, and NK cells activated exhibited the increased infiltration levels in high TTC39C expression group (**Figure 8O**). The ssGSEA results uncovered the increased infiltrations of APC co-inhibition, CD8+ T cells, inflammation-promoting, MHC class I, NK cells, T cell co-stimulation, Tfh, and Th1 cells in high CDKN2A expression group (**Figure 8P**). PKIB upregulation was in relation to most immune functions and immune cell infiltrations (**Figure 8Q**). In **Figure 8R**, we noticed the prominent interactions of high TTC39C expression with activation of most immune functions and immune cell infiltrations. We also estimated the associations of CDKN2A, PKIB, and TTC39C with immune checkpoint molecules. Most immune checkpoint molecules exhibited positive interactions with CDKN2A, PKIB, and TTC39C (**Figures 8S–U**).

## Discussion

AS, a crucial post-transcriptional modification, can produce diverse mRNA variants, which results in structural transcription variation and protein diversity (38, 39). Emerging evidence suggests the functions of AS events in lung carcinogenesis (40). For instance, diverse splicing types of regulators of cell apoptosis may affect NSCLC progression through modulating the imbalance between pro-apoptosis and apoptosis (41–43). Herein, we systematically uncovered the prognostic implications and immunity of AS events in LUAD.

Herein, in total, 43,945 AS events were identified across LUAD, indicating that AS might be a common modification in LUAD. Following survival analysis, we observed 2415 OS-related AS events as well as distinct splicing types had specific splicing preferences, which might assist in formulating more effective treatment regimens. Previous studies have shown that the binding of splicing factors to specific RNA sequences in genome determines precise regulation of RNA splicing (44). Thus, an integrative analysis was conducted for addressing the underlying mechanisms involving them during lung tumorigenesis. The OS-relevant splicing factor-AS interaction network showed the prominent interactions of 44 OS-relevant AS events with three splicing factors (SEC31B, CLK1, and DDX39B). Previously, CLK1 could modulate the chemoresistance of glioma cells *via* glycolytic signaling mediated by AMPK/mTOR/HIF-1α (45) as well as participating in modulating the splicing process of gastric cancer, serving as an underlying therapeutic target against this malignancy (46). Chemical suppression of CLK1 may disrupt the recruitment of internal kinetochores as well as impair cell cycle progression, contributing to unprogrammed cell death (47). Moreover, inhibition of DDX39B triggers sensitivity of BRCA1-mutant ovarian cancer cells to chemotherapy drugs such as platinum and PARPi (48). Our data indicate that splicing factors and AS events were not only one-to-one coordination or antagonistic regulatory interactions, revealing the complexity of their regulatory network.

With the LASSO method, we established an AS event-based prognostic model (BEST3|23330|AT, CDKN2A|86004|AP, TTC39C|44852|AP, MEGF6|315|ES, PKIB|77377|AP, CA5B|98313|ES, HNRNPLL|53258|AT, LDB1|12935|AP, C12orf76|24406|AT, AP2B1|40327|AD, LETM2|83398|AT, MRPL33|53046|ES) in LUAD. In-depth analysis verified that this model could accurately indicate outcomes of LUAD patients. Accumulated evidence suggests that AS events are in relation to the remodeling of the tumor microenvironment (15, 49). Herein, our data uncovered the high-risk group presented the features of decreased infiltrations of immune and stromal cells as well as increased tumor purity. Additionally, LUAD patients with a high risk presented worse immune reactivity, which might contribute to shorter survival duration as well as higher degree of malignancy. TMB was characterized as an effective indicator for prediction of clinical response to immunotherapy (34, 50, 51). Our data indicated that the high-risk group presented higher TMB score, which revealed that patients in high-risk groups may experience better outcomes with immunotherapy. Subgroup analysis uncovered those patients with reduced TMB score and increased risk score tended to exhibit more malignant clinical outcomes and shorter survival duration. Moreover, we noticed that patients with a high-risk score presented higher priority to cisplatin, gemcitabine, erlotinib, and gefitinib, providing a reference for the choice of the optimal chemotherapeutic or targeted therapeutic regimen.

Previous research revealed the parental genes of AS events displayed deregulation owing to abnormal AS events (52). Therefore, we identified 12 AS-relevant genes (BEST, CDKN2A, TTC39C, MEGF6, PKIB, CA5B, HNRNPLL, LDB1, C12orf76, AP2B1, LETM2, MRPL33) in the AS event-based prognostic model. Further, we investigated the upregulation of CDKN2A, TTC39C, and PKIB expressions in LUAD as well as their upregulation was indicative of dismal outcomes in LUAD. Further analysis uncovered that highly expressed PKIB was related to increased infiltrations of immune and stromal cells and opposite findings were investigated for TTC39C. Additionally, CDKN2A, TTC39C, and PKIB exhibited positive associations with most immune checkpoint molecules across LUAD. The data indicated that CDKN2A, TTC39C, and PKIB exerted critical functions in modulating tumor immunity of LUAD. Previously, CDKN2A was shown to be associated with polymorphism of GSTs genes in esophageal squamous cell carcinoma (53). PKIB facilitates breast and lung carcinogenesis through modulating Akt signaling (54). To date, TTC39C has no relevant literature reports on its role in tumorigenesis. Several limitations have been pointed out in our study. Firstly, the AS event-based prognostic model was developed based on a retrospective cohort. The predictive power of this model needs to be validated in more prospective cohorts. Moreover, the limited evidence is not enough to fully explain the specific roles of these genes in lung tumorigenesis. In follow-up studies, we will conduct further experiments to validate our findings.

## Conclusion

Collectively, our research presented systematic analyses of AS events across LUAD, and finally developed a reliable and independent prognostic model on the basis of AS events. Our in-depth analyses revealed the interactions of AS events with immune response and immune cell infiltrations. Finally, we identified three prognostic AS-event-related genes that might play a non-negligible role in lung carcinogenesis. Nevertheless, their potential significance as prognostic indicators and therapeutic targets in clinical applications deserve further study.

## Data Availability Statement

The datasets presented in this study can be found in online repositories. The names of the repository/repositories and accession number(s) can be found in the article/**Supplementary Material**.

## Ethics Statement

The studies involving human participants were reviewed and approved by Ethics committee of China Medical Uni. The patients/participants provided their written informed consent to participate in this study. Written informed consent was obtained from the individual(s) for the publication of any potentially identifiable images or data included in this article.

## Author Contributions

Author GB and YX performed the statistical analyses and wrote the manuscript. Author GB completed all of the data entry and provided assistance for the data analysis. Author GB, XG, YY, JL, and XZ were responsible for the diagnosis and clinical assessment of the participants. Author XZ and TL designed and wrote the study protocol and reviewed the manuscript. Author XG participated the revision of this manuscript. In addition, author YY, YX, and JL offered many constructive opinions on this study and provided a critical revision of the manuscript for important intellectual content. All authors contributed to and approved the final manuscript.

## Funding

This work was supported by Wu Jieping Medical Foundation (320.6750.2020-17-7).

## Conflict of Interest

The authors declare that the research was conducted in the absence of any commercial or financial relationships that could be construed as a potential conflict of interest.

## Publisher’s Note

All claims expressed in this article are solely those of the authors and do not necessarily represent those of their affiliated organizations, or those of the publisher, the editors and the reviewers. Any product that may be evaluated in this article, or claim that may be made by its manufacturer, is not guaranteed or endorsed by the publisher.

## Supplementary Material

Supplementary Figure 1 Correlations between prognostic model and immune cell infiltrations across LUAD, including **(A)** B cells naïve, **(B)** dendritic cells resting, **(C)** mast cells resting, **(D)** monocytes, **(E)** T cells CD4 memory resting, **(F)** macrophage M0, **(G)** macrophage M1, **(H)** NK cells resting, **(I)** T cells CD4 memory activated, **(J)** T cells follicular helper, and **(K)** Tregs.

## Abbreviations

NSCLC, non-small cell lung cancer; LUAD, lung adenocarcinoma; AS, alternative splicing; PSI, Percent Spliced In; AD, Alternate Donor site; AA, Alternate Acceptor site; AT, Alternate Terminator; AP, Alternate Promoter; ME, Mutually Exclusive Exons; ES, Exon Skip; RI, Retained Intron; OS, overall survival; LASSO, least absolute shrinkage and selection operator; ROC, receiver operating characteristic; ssGSEA, single-sample gene set enrichment analysis; CIBERSORT, Cell type Identification By Estimating Relative Subsets Of RNA Transcripts; ESTIMATE, Estimation of Stromal and Immune Cells in Malignant Tumors using Expression Data; TMB, Tumor mutational burden; IC50, half-maximal inhibitory concentration; AUC, area under the curve.
