# Supplementary figures and images for: Development of a Prognostic Alternative Splicing Signature Associated With Tumor Microenvironment Immune Profiles in Lung Adenocarcinoma
Source: Front Oncol. 2022 Jun 27;12:880478. doi: 10.3389/fonc.2022.880478 (PMC9271776; doi:10.3389/fonc.2022.880478)

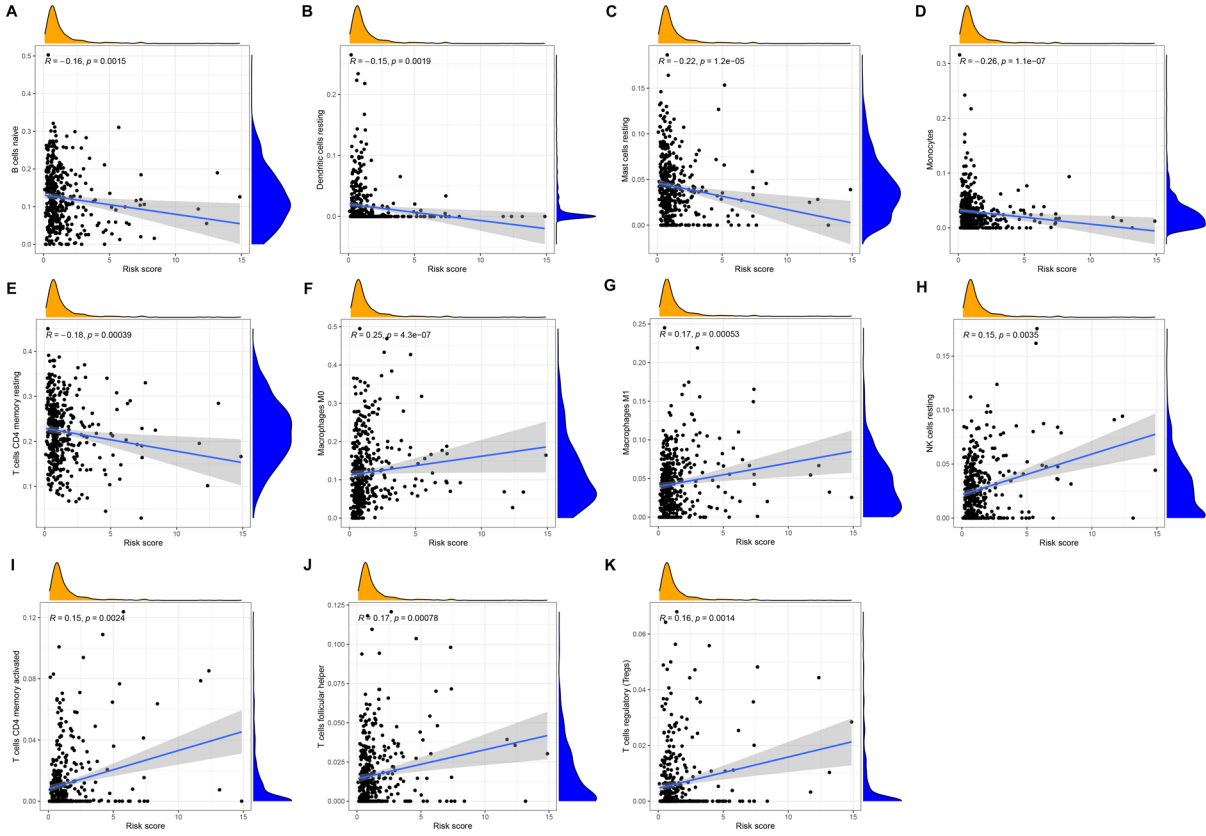

Supplement: Supplementary Figure 1 — Correlations between prognostic model and immune cell infiltrations across LUAD, including (A) B cells naïve, (B) dendritic cells resting, (C) mast cells resting, (D) monocytes, (E) T cells CD4 memory resting, (F) macrophage M0, (G) macrophage M1, (H) NK cells resting, (I) T cells CD4 memory activated, (J) T cells follicular helper, and (K) Tregs. [file Image_1.pdf]
